# Reflecting on Decades of Data: The Global Burden of Disease–Cochrane Project

**DOI:** 10.2196/41323

**Published:** 2024-01-05

**Authors:** Madeline Adelman, Isaac Weber

**Affiliations:** 1 Department of Dermatology University of Colorado Aurora, CO United States; 2 Mercy Hospital St. Louis St. Louis, MO United States

**Keywords:** Global Burden of Disease, Cochrane Library, review, trachoma, onchocerciasis, vitamin A deficiency, data, glaucoma, macular degeneration, vision loss, disorders, disease burden

## Introduction

The Global Burden of Disease (GBD) 2010 study was a systemic epidemiological collaboration between seven institutions to quantify health loss due to diseases, injuries, and risk factors [1]. Its purpose was to develop a platform to compare the magnitude of these health metrics across age groups, countries, sexes, and times, producing comparative metrics for hundreds of causes of premature death and disability. Participating institutions included the “Institute for Health Metrics and Evaluation as the coordinating center, the University of Queensland School of Population Health, the Harvard School of Public Health, the Johns Hopkins Bloomberg School of Public Health, the University of Tokyo, Imperial College London, and the World Health Organization (WHO)” [1].

This project set out to broadly expand the previous GBD 1990 study, conducted primarily by researchers at the World Health Organization and Harvard, to include nearly 500 experts from around the world [2]. In addition, it generated estimates for more than double the number of diseases and sequelae, and improved methods for estimating disability weights. GBD 2010 resulted in estimated disease risk factors, morbidity, and mortality for 291 diseases and injuries and 1160 sequelae [2].

The Cochrane Database of Systematic Reviews (CDSR) is the leading resource for systemic reviews in health care. The GBD-Cochrane project works to assess the representation of different conditions studied in GBD 2010 within CDSR and determine if CDSR accurately reflects GBD disability-adjusted life year metrics.

## Methods

The GBD 2010 study used all available data on cause of death from 187 countries; this included data on vital registration, verbal autopsy, mortality surveillance, censuses, surveys, hospitals, police records, and mortuaries. This data was used to quantify disease burden, disability-adjusted life years, and years of life lost to premature mortality [3].

The GBD-Cochrane project maps the cause-specific disease burden as established by the GBD study to associated systematic reviews of interventions evaluating the same diseases in CDSR. There are seven completed GBD-Cochrane projects and three active projects [4].

## Results

These projects provide high-quality data on systematic reviews and help determine if they poorly or strongly correlate with disease burden. For example, a review of ophthalmologic conditions showed that trachoma, onchocerciasis, vitamin A deficiency, and refraction and accommodation disorders were all underrepresented in the CDSR, while glaucoma, macular degeneration, and other vision loss disorders were overrepresented [5]. Other completed projects have shown poor representation of tropical diseases, while mental health and behavioral conditions are overrepresented [6,7].

## Discussion

There are a plethora of reasons a condition might be overrepresented in the CDSR. Overrepresentation might reflect the high prevalence of these conditions and, therefore, greater availability for randomized clinical trials. Alternatively, overrepresentation may reflect a disparity in funding, the disparity in research in high- versus low-income countries, or the prioritized interest of the public and pharmaceutical companies. Underrepresentation may reflect a decreasing disease burden, existing effective interventions for those conditions, or a lack of researchers in low- and middle-income nations where certain conditions are more prevalent.

The active GBD-Cochrane projects include conditions in the realm of heart disease, cancer, and infectious disease. As the GBD-Cochrane project continues to map systematic reviews and protocols against disease burden, we will continue to identify research gaps and opportunities to make informed decisions with future research.

## Abbreviations

CDSR: Cochrane Database of Systematic Reviews
GBD: Global Burden of Disease
